# Recognizing schizophrenia using facial expressions based on convolutional neural network

**DOI:** 10.1002/brb3.3002

**Published:** 2023-04-16

**Authors:** Xiaofei Zhang, Tongxin Li, Conghui Wang, Tian Tian, Haizhu Pang, Jisong Pang, Chen Su, Xiaomei Shi, Jiangong Li, Lina Ren, Jing Wang, Lulu Li, Yanyan Ma, Shen Li, Lili Wang

**Affiliations:** ^1^ Department of Psychiatry Tianjin Anding Hospital, Mental Health Center of Tianjin Medical University Tianjin China; ^2^ Institute of Mental Health Tianjin Anding Hospital, Mental Health Center of Tianjin Medical University Tianjin China

**Keywords:** clinical clues, convolutional neural network, facial expressions, schizophrenia

## Abstract

**Objective:**

Facial expressions have been served as clinical symptoms to convey mental conditions in psychiatry. This paper proposes to recognize patients with schizophrenia (SCZ) using their facial images based on deep learning algorithm, and to investigate objective differences in facial expressions between SCZ patients and healthy controls using deep learning algorithm and statistical analyses.

**Methods:**

The study consists of two parts. The first part recruited 106 SCZ patients and 101 healthy controls, and videotaped their facial expressions through a fixed experimental paradigm. The video data were randomly divided into two sets, one for training a convolutional neural network (CNN) with the classification of “healthy control” or “SCZ patient” as output and the other for evaluating the classification result of the trained CNN. In the second part, all facial images of the recruited participants were put into another CNN separately, which was priorly trained with a facial expression database and will output the most likely facial expressions of the recruited participants. Statistical analyses were performed on the obtained facial expressions to find out the objective differences in facial expressions between the two recruited groups.

**Results:**

The trained CNN achieved an overall accuracy of 95.18% for classifying “healthy control” or “SCZ patient.” Statistical results on the obtained facial expressions demonstrated that the objective differences between the two recruited groups were statistically significant (*p* < .05).

**Conclusions:**

Facial expressions hold great promise as SCZ clues with the help of deep learning algorithm. The proposed approach would be potentially applied to mobile devices for autorecognizing SCZ in the context of clinical and daily life.

## Graphical abstract

A deep learning approach is used to recognize the facial expressions of schizophrenia (SCZ) patients, which would be potentially applied to mobile devices for auto‐recognizing SCZ in the context of clinical or/and daily life. Differences in facial expressions between healthy controls and SCZ patients using the second CNN were significant.

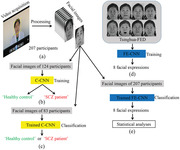

## 1 INTRODUCTION

Schizophrenia (SCZ) is a debilitating mental disorder characterized clinically by a variety of psychotic symptoms, including hallucinations, delusions, disorganized speech, and disorganized or catatonic behavior (Farah, 2018; Lammer et al., 2018). Approximately 1.30% of the population worldwide suffers from SCZ, which affects the individual's ability to meaningfully engage in daily activities and maintain relationships (Farooq et al., 2016; Mccutcheon et al., 2020). Because there are still no reliable or robust biomarkers, SCZ is more difficult to diagnose than physical disorders. The major challenges in the diagnosis of SCZ or other psychiatric disorders are heterogeneity and nonspecificity (Davison et al., 2018; Rodrigues‐Amorim et al., 2017). Till now, the diagnosis of SCZ still relies exclusively on the potentially subjective evaluation of clinical symptoms and social functioning from a psychiatrist, as no clear organic indicators have been identified that would help to make precise judgments and individualized treatment. Due to the lack of quantitative standards and the reliance on subjective evaluation from psychiatrists, the misdiagnosis of SCZ occurs occasionally, resulting in delayed treatment. Therefore, it is crucial to improve the aid–diagnosis of SCZ.

In recent years, there has been a rapidly growing interest in exploring the facial expressions of patients with SCZ or other psychosis (Gao et al., 2021; Veronica Romero‐Ferreiro et al., 2015; Weiss et al., 2009). Facial expressions, which indicate communication cues through facial movements, have been shown to represent emotional states and convey mental conditions as clinical symptoms. In a series of studies, researchers have employed the well‐known Facial Action Coding System (FACS) to convert the action units’ descriptive scores into seven emotions, including “surprised,” “contemptuous,” “fearful,” “happy,” “angry,” “sad,” and “disgusted” (Martinez et al., 2019). A previous study has demonstrated that SCZ patients often exhibit uncertainty in facial expressions compared to healthy controls (Sevos et al., 2018). Moreover, it has been reported that SCZ patients exhibit incongruent facial expressions and are significantly worse than healthy controls in their responses to emotional stimuli and during social interactions (Bersani et al., 2013). Overall, it is still unclear (1) whether SCZ patients could be recognized using their facial expressions and (2) whether there are objective differences in facial expressions between SCZ patients and healthy controls.

For dealing with the above two issues, the identification of facial expressions of SCZ patients and healthy controls is the prerequisite. Although the use of FACS has been already available for identifying facial expressions, there are several difficulties in applying FACS in clinical settings, such as inconvenience, low reproducibility, and time consumption. In addition, it requires professional training (Martinez et al., 2019). Considering the limitation, deep learning offers a unique opportunity to encourage the collection of unprecedented clinical data from a wider range of sources than ever before (Alhasan, 2021; Hwang et al., 2018; Litjens et al., 2019; Zeng et al., 2020). This brings new dimensions to clinical research and brings convenience to clinicians. Among many deep learning algorithms, the convolutional neural network (CNN) highlights the advantages of high accuracy in image recognition and classification (Alhasan, 2021). The advantages of CNN are as follows: (1) it is able to preserve spatial properties of images due to their highly parameterized and sparsely connected kernels; (2) it learns through labeled images and identifies important features without explicitly specifying them; and (3) it learns a representation of input data as the information flow ascends through multiple layers. In recent years, CNN has been successfully applied to medical image classification, depression detection, and mental health status identification (Chen et al., 2021; Ke et al., 2021; Shafiei et al., 2020). Therefore, the excellent performance of CNN will help to uncover potential clues for recognizing facial expression features of SCZ patients.

In this paper, we propose to recognize SCZ patients using their facial expressions based on CNN and investigate objective differences between the facial expressions of SCZ patients and healthy controls. Our work includes two parts. The first part trained a CNN using facial images of the recruited participants (including healthy controls and SCZ patients), with the classification of “healthy control” or “SCZ patient” as output, which aims at recognizing SCZ patients using their facial expressions. The second part aims at investigating objective differences between the facial expressions of SCZ patients and healthy controls. To do this, all facial images of the recruited participants were separately put into another CNN, which was priorly trained with the Tsinghua facial expression database (Tsinghua‐FED) that will output the most likely facial expressions of the participants (Yang et al., 2020). Statistical analyses were performed on the obtained facial expressions to demonstrate the objective differences between the two recruited groups. In addition, correlation analyses were conducted to evaluate the relationships between SCZ clinical parameters and experimental results. We expect the findings of the study would be useful for SCZ research, and facial expressions could be represented as clinical clues for the aid–diagnosis of SCZ or other mental disorders.

## 2 MATERIALS AND METHODS

### 2.1 Participants

We recruited a total of 106 chronic SCZ patients (male/female: 56/50; average age: 45.05 ± 11.57 years) and 101 age‐ and sex‐matched healthy controls (male/female: 47/54; average age: 42.41 ± 10.95 years) for this study. All SCZ patients were from the inpatient wards of Tianjin Anding Hospital and were screened and evaluated by two experienced psychiatrists independently. The inclusion criteria were as follows: 20–60 years old; SCZ diagnosis according to DSM‐IV criteria via the Structured Clinical Interview (SCID); a stable dose of antipsychotic medication for more than 1 year prior to the enrollment; and Han Chinese. The exclusion criteria included severe physical diseases; organic brain disease; substance abuse other than tobacco; pregnant or lactating women; and language‐related problems. The psychopathology of the SCZ patients was assessed using the Positive and Negative Syndrome Scale (PANSS) (Kay et al., 1987). All healthy controls were recruited from the local community in Tianjin, and none of them had mental disorders or a family history of mental disorders, physical diseases, and language‐related problems.

This current study was approved by the Ethics Committee of Tianjin Anding Hospital, China. All participants signed an informed consent form before participating in the study.

### 2.2 Overview of the proposed approach

**FIGURE 1:**
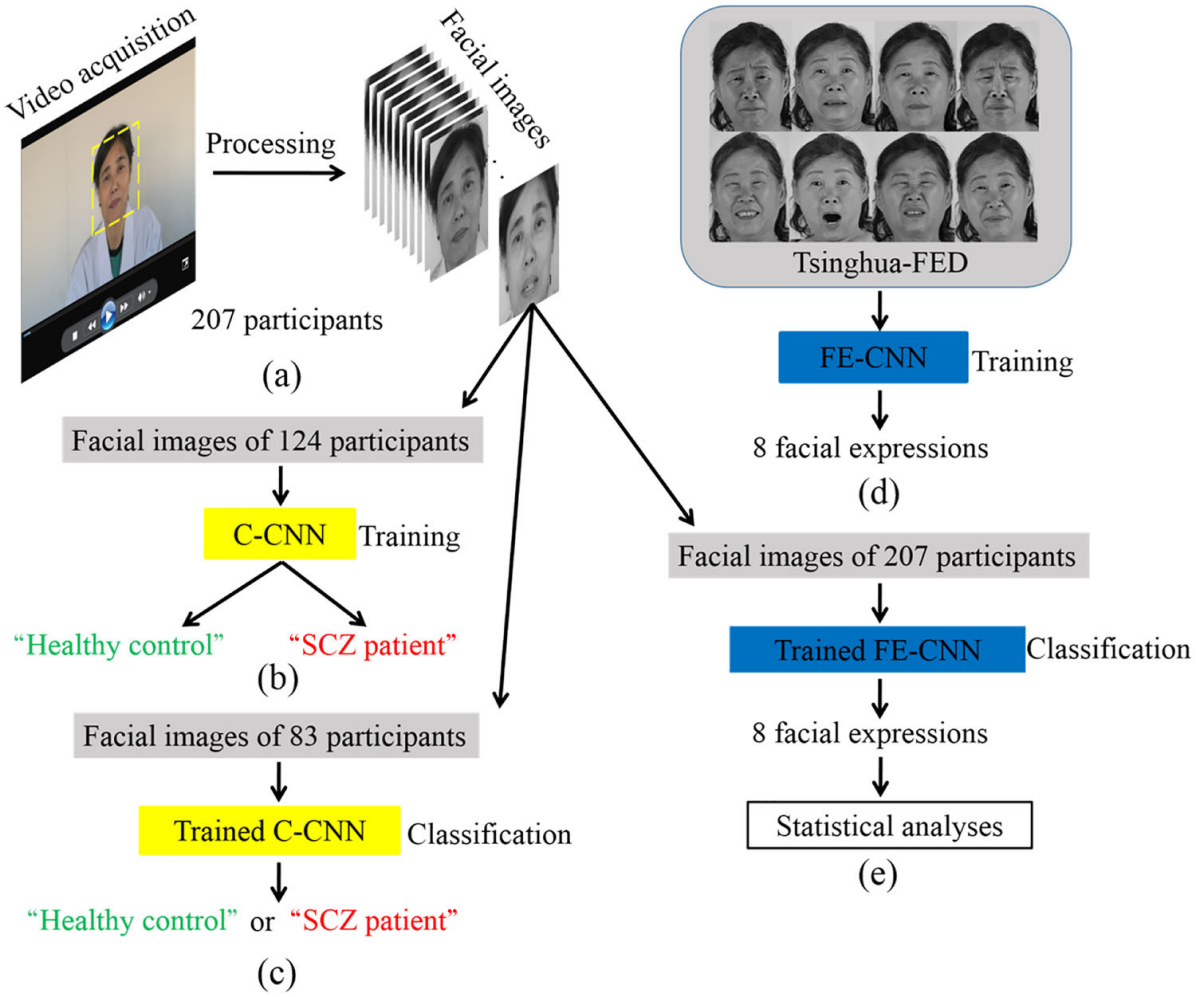
Flow of the proposed approach: (a) video acquisition and processing, (b) facial images of the 124 participants as training data for training C‐CNN, (c) facial images of the 83 participants as testing data for evaluating classification result of the trained C‐CNN, (d) training FE‐CNN with Tsinghua‐FED, and (e) investigate differences between facial expressions of healthy controls and schizophrenia (SCZ) patients with the trained FE‐CNN based on statistical analyses.

Figure 1 shows the flow of the proposed approach. Overall, there are two parts. In the first part, after video acquisition by a fixed experimental paradigm, a series of two‐dimensional gray facial images of the participants were processed, as shown in Figure 1a. Then, the participants were divided into two sets randomly—a training set and a testing set. There were 124 participants in the training set (including 62 healthy controls and 62 SCZ patients) and 83 participants in the testing set (including 39 healthy controls and 44 SCZ patients). Facial images of the 124 participants in the training set were considered training data for training a CNN, named C‐CNN, with the classification of “healthy control” or “SCZ patient” as output, as shown in Figure 1b. Facial images of the 83 participants in the testing set were regarded as testing data that were used to evaluate the classification results of the trained C‐CNN, as shown in Figure 1c.

In the second part, all facial images of the participants were separately put into another CNN shown in Figure 1d, named FE‐CNN. The FE‐CNN was priorly trained with Tsinghua‐FED that will output the most likely facial expressions of the participants, as shown in Figure 1e. Then, statistical analyses were performed on the obtained facial expressions to find out the objective differences in facial expressions between SCZ patients and healthy controls.

### 2.3 Video acquisition and processing

A fixed experimental paradigm was designed, including six specified questions (stimuli) presented in Table 1. The six specified questions were determined according to a relevant study measuring depression symptom severity from facial expressions with several commonplace questions (Haque et al., 2018). A smart speaker directly connected to a computer was employed to play the six questions. Video acquisition was conducted in a fixed conference room at Tianjin Anding Hospital. A matte white wall was used as the background. All participants were attired in uniform white coats, with no glasses and no colored makeup; they were informed there were no correct or false answers; and their responses would be kept strictly confidential. Each participant completed the experimental paradigm alone for approximately 3 min. A digital camera (LX10, Panasonic, Japan) with 50 frames per second and 1920 × 1080 pixels was placed approximately 150 cm in front of each participant's face to videotape their facial expressions.

**TABLE 1:** The six specified questions involved in the experimental paradigm.

	Questions (stimuli)
1	Please do a self‐introduction.
2	What makes you happy recently?
3	Did you disgust someone spoke evil of you behind your back？
4	Who is the one you are anxious to see? Please give a reason.
5	What makes you sad recently?
6	Are you scared to confront the camera or psychiatrists?

A total of 207 videos were saved and archived according to their labels (“healthy control” or “SCZ patient”). For each participant, six time slots with regard to answering the six specified questions were recorded. Video processing was implemented in MATLAB software (2019a, MathWorks, USA). First, each video was converted to a two‐dimensional gray photo sequence. Then, numerous photos were spun off from the photo sequences under the six time slots. Finally, facial images involved in these photos were extracted using the Viola–Jones algorithm and were resized to 320 × 240 pixels (Viola & Jones et al., 2004).

### 2.4 Tsinghua‐FED

We received the approval to use Tsinghua‐FED for this study, from the Tsinghua‐FED team in Sun's Lab, Department of Psychology at Tsinghua University, China. The Tsinghua‐FED consists of 880 color facial images of 110 Chinese young and old adults displaying eight facial emotional expressions, namely “neutral,” “happy,” “angry,” “disgusted,” “surprised,” “fearful,” “content,” and “sad.” All color facial images have been validated by the Tsinghua‐FED team and stored in JPG format with 1800 × 2200 pixels (Yang et al., 2020).

### 2.5 Architectures of C‐CNN and FE‐CNN

**FIGURE 2:**
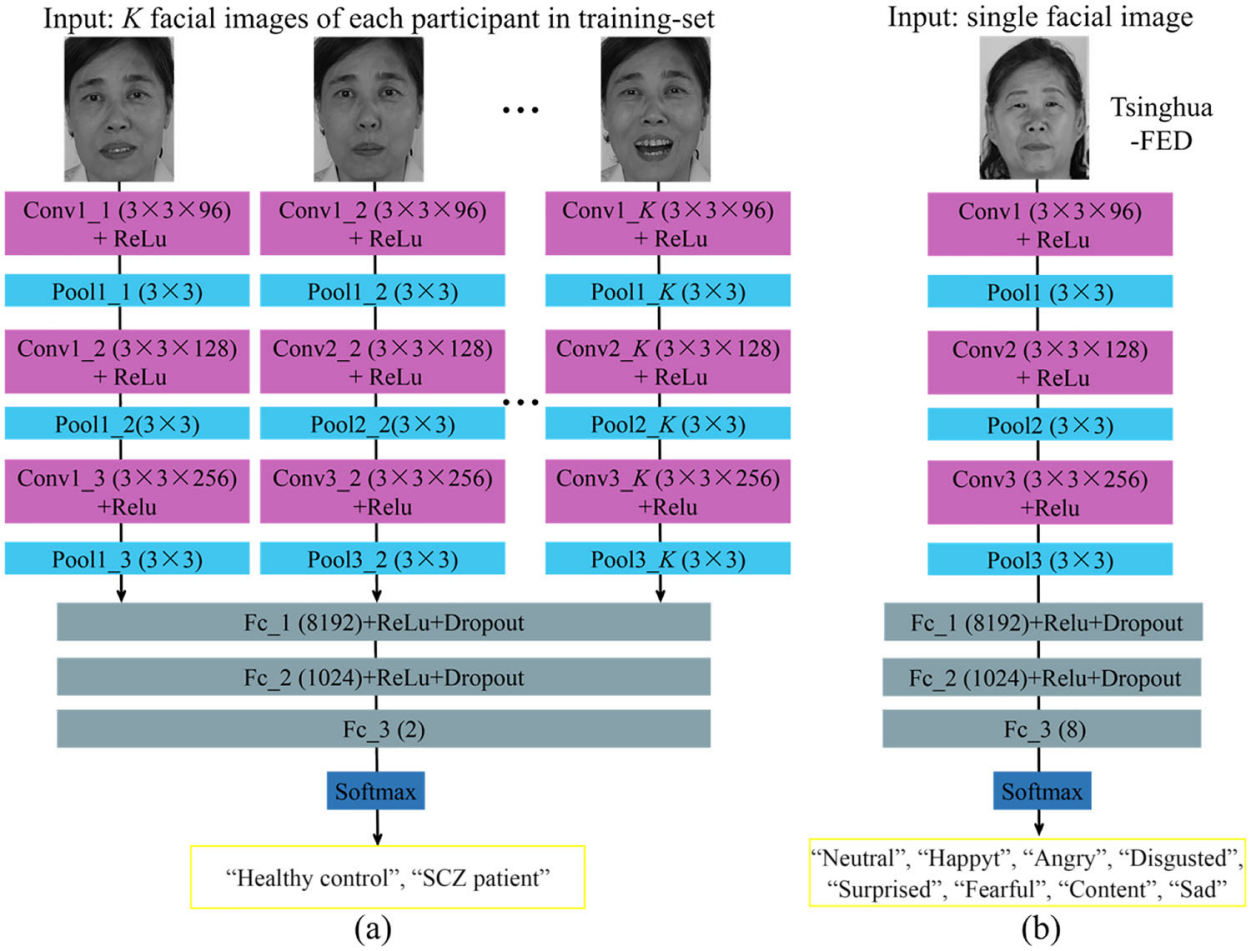
Architectures of (a) C‐CNN and (b) FE‐CNN. Relu, Dropout, and Softmax denote activation function, dropout regularization, and softmax classifier. In C‐CNN, Conv*j_k* and Pool*j_k* denote the *k* (*k* = 1, 2, … *K*) th facial image passed the *j* (*j*= 1, 2, 3) th convolutional and max‐pooling layers; Fc_*p*(*q*1) denotes the *p* (*p* = 1, 2, 3) th fully connected layer with *q*1 (*q*1 = 8192, 1024, 2) nodes. In FE‐CNN, Conv*j* and Pool*j* denote the single facial image passed the *j* (*j*= 1, 2, 3) th convolutional and max‐pooling layers; Fc_ *p*(*q*2) denotes the *p*th fully connected layer with *q*2 (*q*2 = 8192, 1024, 8) nodes. Both in C‐CNN and FE‐CNN, suffixes (3 × 3 × *m*) and (3 × 3) denote the convolutional layer with *m* (*m* = 96, 128, 256) filters of a spatial size 3 × 3 and the max‐pooling layer of a spatial size 3 × 3.

Both C‐CNN and FE‐CNN were implemented by using the deep learning toolbox of MATLAB software. Figure 2 presents the architecture of C‐CNN and FE‐CNN. For C‐CNN, we used *K* facial images of each participant in the training set as the training data. The *K* facial images of each participant were extracted from the six time slots (i.e., *K*/6 facial images were extracted at each time slot with equal intervals). We adopted the late fusion strategy to C‐CNN to achieve strong learning performance (Zheng et al., 2019). Accordingly, *K* facial images passed through six feature maps with shared parameters, and then *K* streams were merged in the first fully connected layer. The final fully connected layer was connected to a softmax classifier to output the classification of “healthy control” or “SCZ patient.” The softmax classifier, one of the most important operators in deep learning, normalizes various features according to the number of classifications and generates a probability distribution for each classification (Djavanshir et al., 2021). Accordingly, C‐CNN selects the classification with the maximum probability as the output. For FE‐CNN, the input was a single facial image from Tsinghua‐FED. All color facial images from Tsinghua‐FED were converted to two‐dimensional gray facial images and were resized to 320 × 240 pixels before being input to FE‐CNN. The input of FE‐CNN also passed through six feature maps and then connected to three fully connected layers and a softmax classifier to output the classification of eight facial expressions.

### 2.6 C‐CNN and FE‐CNN training settings

In model training work, a batch size of 3 and an initial learning rate of 0.0001 were set. All convolutional and max‐pooling layers were padded to zero with a fixed 2 × 2 stride region. A fixed dropout rate of 0.5 was attached to prevent overfitting. We utilized the gradient descent method to adjust model parameters after each iteration (Zheng et al., 2019), so as to achieve the optimum model parameters through multiple iterations.

To achieve a better training performance on small data and also to further prevent the overfitting problem, we applied a data augmentation strategy to the training data and the Tsinghua‐FED (Nanni et al., 2021). The data augmentation strategy has been proven to be beneficial to the generalization ability and robustness of deep learning algorithms. In particular, rotation augmentation has been shown to be the most efficient data augmentation strategy (Nanni et al., 2021). Accordingly, all facial images in our training data and Tsinghua‐FED were rotated by 180 degrees to create new facial images; thus, the number of facial images in our training data and Tsinghua‐FED was doubled in this way.

### 2.7 Statistical analyses

All data analyses, model training, and experiments were run on a standard workstation (64 GB RAM, 3.70 GHz Intel Core i9 CPU, NVidia Quadro P6000, 56 GB VRAM). The SPSS software (20.0, IBM Corporation, USA) was used to perform statistical analyses. First, a Kolmogorov–Smirnov one‐sample test was used to determine whether the data were normally distributed. The data with normal distribution were expressed as mean differences ± standard deviation ($\bar{x}\pm s$), and a *t*‐test was used for comparison between groups. Statistical data that were not normally distributed were expressed as median, and the Wilcoxon rank‐sum test was employed to compare between groups. The count data were expressed as frequencies, and the *χ* ^2^ test was employed for comparison between groups. The significance level for two tails was set at *p* = .05.

## 3 RESULTS

### 3.1 General information of the participants

Table 2 presents the demographic and clinical data of the participants, and compares the statistical differences between SCZ patients and healthy controls. As can be seen, there were no significant between‐group differences in age, sex, and education (all *p*s > .05).

**TABLE 2:** Demographic and clinical data of schizophrenia (SCZ) patients and healthy controls.

Variables	SCZ patients (*n* = 106)	Healthy controls (*n* = 101)	*t*/*χ* ^2^	*p*
Age (years)	45.05 ± 11.57	42.41 ± 10.95	–1.69	0.09
Sex (male /female)	56 / 50	47 / 54	0.82	0.37
Education (years)	11.15 ± 3.36	11.71 ± 4.93	0.96	0.34
PANSS total score	80.19 ± 16.67	–	–	–
Positive subscore	20.20 ± 7.21	–	–	–
Negative subscore	23.63 ± 6.14	–	–	–
General psychopathology	36.32 ± 8.26	–	–	–

*Note*: Data presented as mean ± standard deviation.

### 3.2 Training processes of C‐CNN and FE‐CNN

Figure S1 shows the loss functions and iteration accuracies of C‐CNN (e.g., *K* = 360) and FE‐CNN in training processes. The loss functions were calculated using the *l*_2_‐norm; the iteration accuracies here especially referred to C‐CNN's or/and FE‐CNN's classification accuracies to the training data and the Tsinghua‐FED. Considering there were no evident changes in loss functions and iteration accuracies after 8000 iterations, as well as the loss functions and iteration accuracies were stably converged at 8000 iterations, all the iterations were terminated at 8000.

### 3.3 Classification to testing data with the trained C‐CNN

**FIGURE 3:**
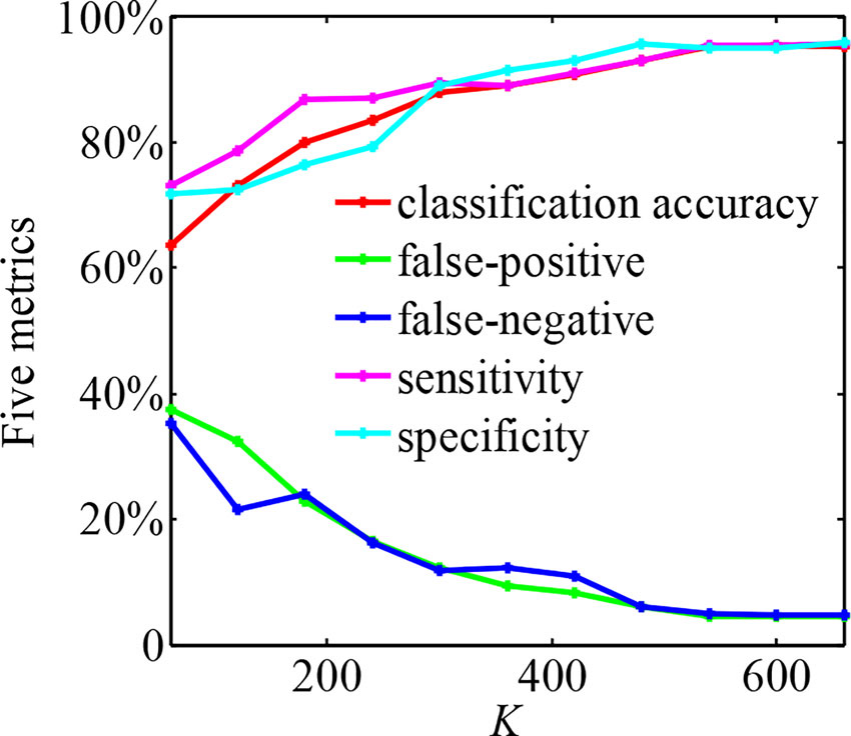
Quantitative evaluations for the classification of testing data with the trained C‐CNN under different amounts of input facial images.

We repeated the C‐CNN training and testing processes five times under different amounts of input facial images (*K* = 60, 120, …, 660) and quantitatively evaluated the classification results using five metrics, namely classification accuracy, false positive, false negative, sensitivity, and specificity (Sefers et al., 2005). Figure 3 plots *K* as functions of the average values of the five metrics five times. As can be seen, C‐CNN's classification accuracy to testing data exceeded 80% when *K* > 120, whereas the reduction in facial images resulted in a substantial decrease in features in the architecture of C‐CNN. In addition, C‐CNN's classification accuracy to testing data increased with the increment of *K*, and there is no improvement in classification results to testing data when *K* > 540. The C‐CNN training process took about 185 h at *K* = 540, and the five metrics were determined to be 95.18%, 5.13%, 4.67%, 95.51%, and 94.90% for classification accuracy, false positive, false negative, sensitivity, and specificity, respectively. The experimental results indicate that the objective differences existed in facial expressions between SCZ patients and healthy controls, and validate the effectiveness of our experimental paradigm.

It is worth noting that the experimental results would be different when using facial images extracted from each individual time slot as input for the C‐CNN training and testing processes. Therefore, for each participant in the training set and testing set, we used their 90 facial images that were extracted at equal time intervals in each individual time slot as training data and testing data for the C‐CNN training and testing processes. We repeated this experiment five times; the average values of the five metrics for the five times are presented in Table 3, which allowed us to separately study the performance of each question (stimulus) to the experimental results. It is seen that using only one specified question showed a weak performance in our experimental results (the classification accuracy ≤76.92%), suggesting that multiple specified questions involved in our experimental paradigm might be critically important. In addition, the C‐CNN trained using facial images at time slot 1 could not achieve high performance to distinguish SCZ patients from healthy controls; as seen in Table 3, the false positive is the largest and the classification accuracy is the lowest at time slot 1, denoting that the use of emotionally meaningful stimulus for our experimental paradigm was superior to the neutral stimulus.

**TABLE 3:** Classification performance for the testing data with the C‐CNN trained with facial images in each individual time slot.

Time slot	Classification accuracy	False positive	False negative	Sensitivity	Specificity
1	62.32%	41.13%	36.32%	71.56%	73.42%
2	72.44%	27.56%	27.64%	78.67%	78.45%
3	74.65%	25.05%	23.38%	79.14%	81.56%
4	76.92%	28.33%	24.54%	80.53%	80.18%
5	70.42%	30.09%	29.21%	76.96%	77.74%
6	71.26%	28.52%	29.44%	77.87%	77.24%

### 3.4 Classification to our own database with the trained FE‐CNN

Considering that the Tsinghua‐FED database and our own database have different experimental conditions, we have evaluated FE‐CNN's classification performance on our own database. In this work, 100 facial images (50 for SCZ patients, 50 for healthy controls) for each facial expression were extracted from our own database and labeled by two psychiatrists. These facial images were separately put into the trained FE‐CNN to determine the most likely facial expressions. We have calculated the confusion matrix with reference to the actual labels from the two psychiatrists, as presented in Table 4, where each row represents the classification labels, and each column represents the actual labels. Apparently, the trained FE‐CNN achieved classification accuracies of ≥80.00 for all facial expressions except “Angry”; the average classification accuracy for the labeled facial images was determined to be 83.38%, demonstrating FE‐CNN's performance to classify our own database.

**TABLE 4:** Confusion matrix for using the trained FE‐CNN to classify the labeled facial images from our own database.

	Neutral	Disgusted	Fearful	Happy	Sad	Surprise	Content	Angry
Neutral	**83.00%**	0.00%	4.00%	1.00%	2.00%	3.00%	7.00%	0.00%
Disgusted	0.00%	**80.0%**	0.00%	8.00%	0.00%	4.00%	8.00%	0.00%
Fearful	8.00%	0.00%	**82.00%**	1.00%	1.00%	7.00%	1.00%	0.00%
Happy	2.00%	2.00%	0.00%	**87.00%**	1.00%	1.00%	7.00%	0.00%
Sad	6.00%	1.00%	3.00%	0.00%	**80.00%**	1.00%	7.00%	2.00%
Surprised	3.00%	0.00%	5.00%	2.00%	0.00%	**89.00%**	1.00%	0.00%
Content	2.00%	0.00%	4.00%	4.00%	1.00%	1.00%	**88.00%**	0.00%
Angry	2.00%	1.00%	4.00%	1.00%	2.00%	1.00%	11.00%	**78.00%**

### 3.5 Differences in facial expressions between SCZ patients and healthy controls

**FIGURE 4:**
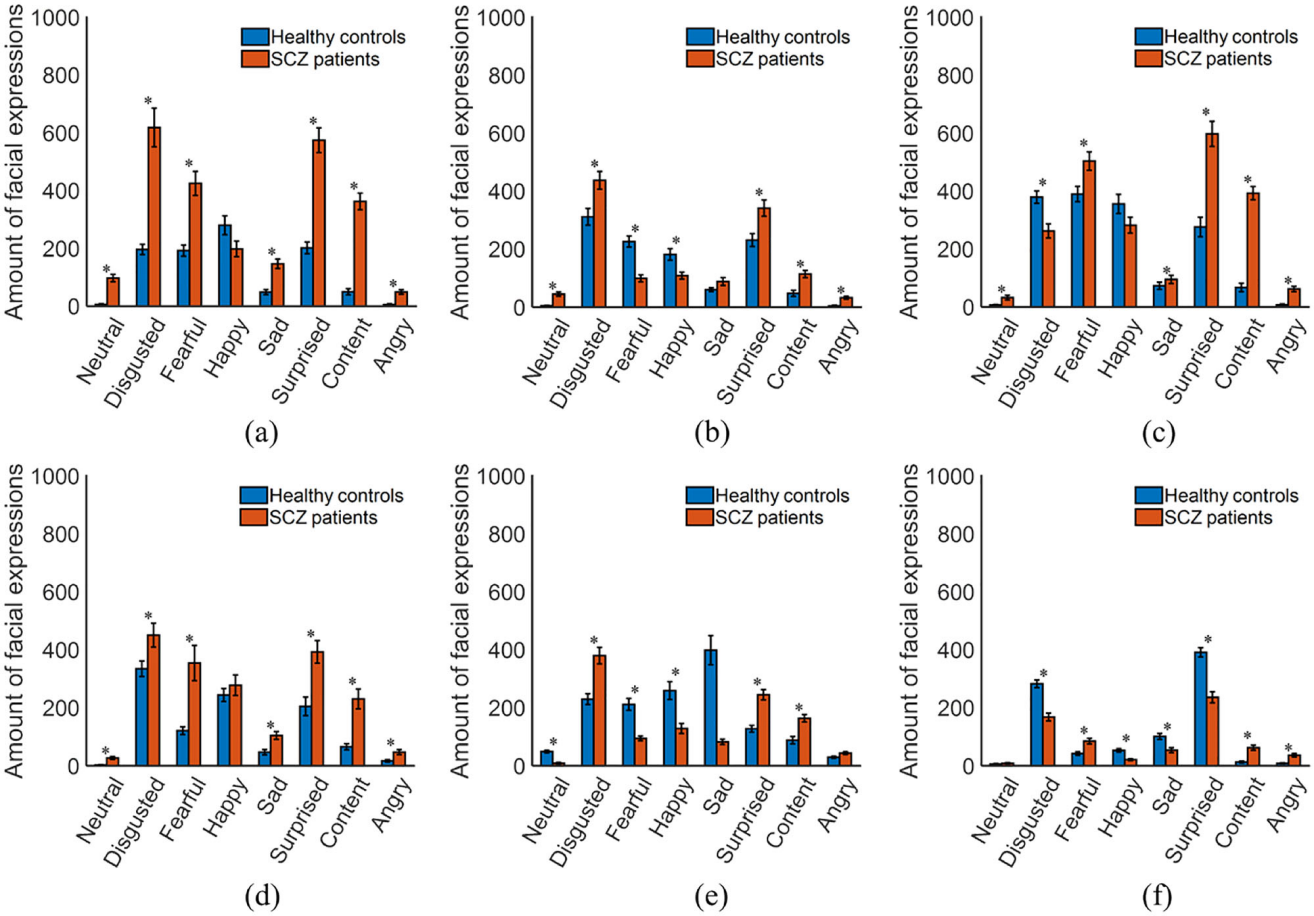
Statistical results for the amount of eight facial expressions of the participants at (a–f) time slot 1 to time slot 6. *p < .05.

**FIGURE 5:**
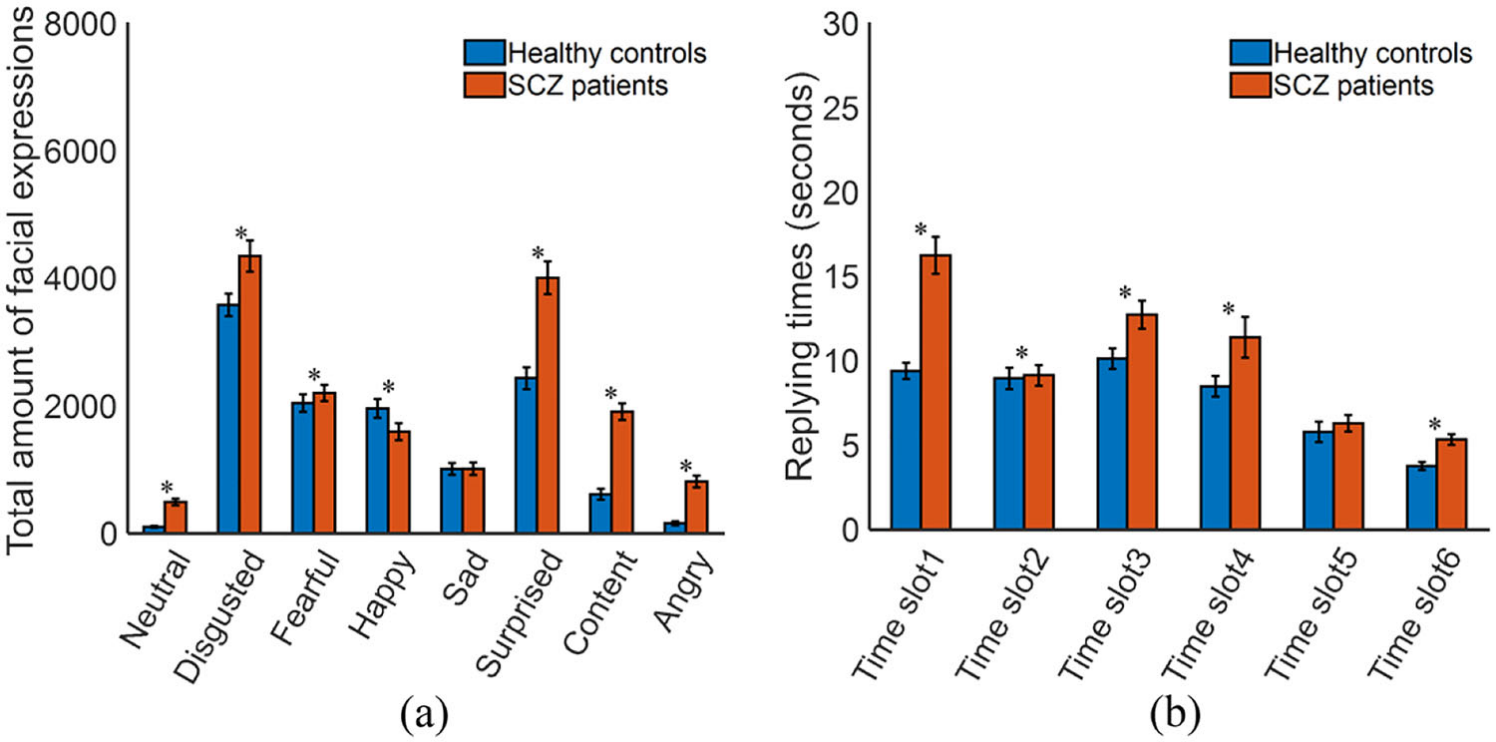
Statistical results for (a) the total amount of eight facial expressions and (b) response times of the participants in terms of the six time slots. *p < .05.

**FIGURE 6:**
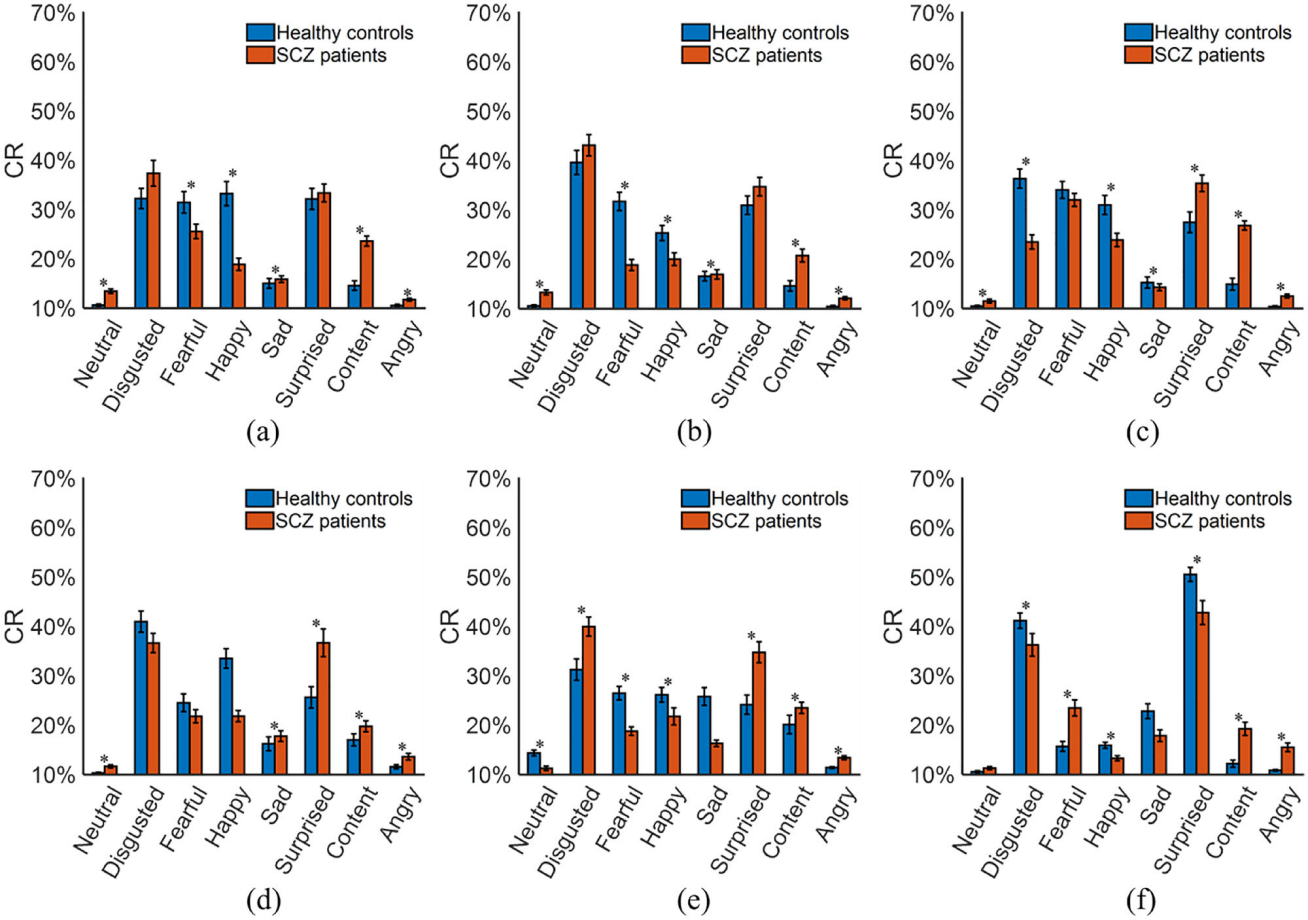
Statistical results for constituent ratio (CR) values of the participants at (a–f) time slot 1 to time slot 6. *p < .05.

All facial images of the participants were individually put into the trained FE‐CNN; the output for each facial image was the most likely facial expression. Statistical analyses were performed in three aspects, including the amount of facial expressions, the response time, and the constituent ratio (CR) of each facial expression. The CR is introduced as CR=Y(i)/Y(i)∑i=18Y(i)∑i=18Y(i), where *Y*(*i*) denotes the amount of the *i* (*i* = 1, 2, …, 8) th facial expressions. Figure 4 shows the statistical results for the amount of eight facial expressions of the participants under the six time slots. Figure 5 presents the statistical results for the total amount of eight facial expressions and the response time (i.e., the duration of each time slot) of the participants. The total response times were 96.94 ± 27.54 s for healthy controls and 126.42 ± 39.75 s for SCZ patients (*p* < .05). Figure 6 shows the statistical results for the CR values of the participants under the six time slots. The statistical results demonstrate that the objective differences in several facial expressions between SCZ patients and healthy controls were statistically significant.

### 3.6 Correlation analyses of SCZ clinical parameters with experimental results

In SCZ group, we performed Pearson correlation analyses between SCZ clinical parameters and total response times (or the CR of each facial expression) during the experimental paradigm. The SCZ clinical parameters include the N1 and total negative symptom score of PANSS, and the duration and the dose of antipsychotic medication (chlorpromazine equivalent). The calculated correlation coefficients and significance coefficients, (*r*, *p*) pairs, are presented in Table 5 (*p* < .05 indicating significance level). As can be seen, SCZ patients’ total response times and CR of each facial expression are not correlated with the SCZ clinical parameters. This might be because the statistical results were depending largely on the experimental paradigm, the general information of SCZ patients (including age, gender, education, underlying disease, and other factors), and the following two uncontrollable factors: (1) several participants may not reveal their real emotions in consideration of protecting their privacy, and thus the statistical results depended largely on participants’ penchant for privacy; (2) composite facial expressions are common in humans, yet the investigation in the study included only eight facial expressions.

**TABLE 5:** The calculated (*r*, *p*) pairs for denoting correlations of clinical parameters with total response times or constituent ratio (CR) of each facial expression during the experimental paradigm.

	N1	Total negative symptom score	Duration	Dose
Total response times	(.05, .53)	(.11, .07)	(.09, .30)	(.11, .41)
CR—neutral	(–.05 .65)	(.03, .70)	(.04, .73)	(–.09, .34)
CR—disgusted	(–.09, .36)	(–.08, .37)	(.21, .29)	(.06, .52)
CR—fearful	(.18, .06)	(.01, .99)	(–.05, .59)	(.06, .51)
CR—happy	(–.02, .79)	(.02, .84)	(.05, .60)	(.14, .15)
CR—sad	(–.12, .21)	(.02, .85)	(–.15, .12)	(.01, .96)
CR—surprised	(.13, .17)	(–.01, .95)	(–.13, .17)	(–.14, .15)
CR—content	(–.06, .54)	(.06, .49)	(.02, .81)	(.09, .33)
CR—angry	(–.11, .27)	(.07, .44)	(–.01, .89)	(–.10, .29)

We also performed Pearson correlation analyses between SCZ clinical parameters and C‐CNN's prediction probability for “SCZ patient” (calculated by softmax classifier). The calculated (*r*, *p*) pairs are presented in Table 6 (*p* < .05 indicating significance level). It is seen that CNN's prediction probability for “SCZ patient” is not associated with any SCZ clinical parameters; the reason account for this has been mentioned above.

**TABLE 6:** The calculated (*r*, *p*) pairs for denoting the correlations of schizophrenia (SCZ) clinical parameters with C‐CNN's prediction probabilities for “SCZ patient.”.

	N1	Total negative symptom score	Duration	Dose
Prediction probabilities	(.22, .15)	(.33, .13)	(.14, .36)	(.31, .14)

## 4 DISCUSSION

We first recognized SCZ patients using their facial images based on a deep learning algorithm, and further combined it with Tsinghua‐FED to investigate objective differences in facial expressions between SCZ patients and healthy controls. The trained C‐CNN achieved an accuracy of 95.18% in classifying SCZ patients and healthy controls (*K* = 540). Statistical results demonstrate that the objective differences in response times and the CR of each facial expression are statistically significant between SCZ patients and healthy controls. These findings suggest that facial expressions could be potentially used as clinical clues to identify SCZ patients with the help of a deep learning algorithm, and there are objective differences in certain facial expressions between SCZ patients and healthy controls (Cowan et al., 2022; Gupta et al., 2022; Hamm et al., 2011).

In SCZ group, C‐CNN's prediction probability for “SCZ patient” and the total response times (or the CR of each facial expression) of SCZ patients during the experimental paradigm are not associated with any SCZ clinical parameters. The reason account for this is the statistical results were depending largely on the experimental paradigm, the general information of SCZ patients, and the two uncontrollable factors mentioned in Section 3.6. Thereby, the next step will do further studies on the optimization of the experimental paradigm, the regression analyses with all information of SCZ patients, and the investigation of composited facial expressions in a larger independent sample.

Considering that the two uncontrollable factors, as well as SCZ patients, often exhibit uncertainty in facial expressions compared to healthy controls (Sevos et al., 2018), it is an open issue to find which facial expression would SCZ patients show more or less than healthy controls, regardless of what the experimental paradigm is and how it is performed. Two alternative strategies will be potentially adopted in our further investigations to find out objective differences in facial expressions between SCZ patients and healthy controls: (1) a probability threshold (such as .3) can be introduced in the output of FE‐CNN, and the classification labels in “softmax2” with probabilities greater than the probability threshold can be considered as the output of FE‐CNN, by which the composite facial expressions can be merged and studied; (2) the CAS(ME)3 database can be introduced for decoding more facial expression types of the participants. (Li et al., 2023). Additionally, an attempt will be made to manually analyze participants’ emotions by extracting their voices, gestures, and facial expression variations from the collected videos.

To the best of our knowledge, there are no fixed questions set as stimuli for measuring psychotics from their facial expressions (Haque et al., 2018), and the experimental paradigm in our study was designed to stimulate facial expressions produced by the participants and compare the objective differences between SCZ patients and healthy controls. The experimental results proved that these differences could be sensed by the use of C‐CNN, whereas the labels of “SCZ patient” and “healthy control” could be classified effectively.

In our study, the ratio of the number of participants in the training set to that in the testing set is about 6:4 (124 participants in the training set, 83 participants in the testing set). To validate the proposed approach further, we have utilized the traditional 7:3 and 10‐fold cross‐validation methods to conduct C‐CNN validations under the same model parameters (dropout rate = 0.5, batch size = 3, initial learning rate = 0.0001) (Baur et al., 2022). Stable convergences in all C‐CNN training processes were achieved at less than 8000 iterations, thus avoiding unexpected overfitting that could confound the experimental results. We repeated the C‐CNN training and testing processes five times for both the traditional 7:3 and the 10‐fold cross‐validation methods; the relationship curves of the calculated average values of the five metrics versus *K* are shown in Figure S2. The results denote that the classification performance of C‐CNN for the testing set increases in proportion to the increment of *K*, and there are no improvements in classification results when *K* > 540. The maximum classification accuracies of the traditional 7:3 and the 10‐fold cross‐validation methods for the testing set were 93.57% and 96.13%, respectively.

As the facial differences between SCZ patients and healthy controls could be sensed by C‐CNN, it is reasonable that the more facial differences sensed by C‐CNN, the better the experimental results. In our experimental paradigm, the first question “Please do a self‐introduction” is a neutral stimulus that may not lead to obvious facial differences between groups, thereby C‐CNN trained using facial images at time slot 1 could not achieve high performance to distinguish SCZ patients from healthy controls; as seen in Table 3, the experimental results denote the superiority of the emotionally meaningful stimulus than neutral stimulus. This also makes more sense, since SCZ patients typically have more emotional problems than healthy controls (Farah, 2018; Lammer et al., 2018), and are significantly worse than healthy controls in their responses to emotional stimuli and during social interactions (Bersani et al., 2013). In addition, the experimental results in Table 3 revealed performance differences among different emotionally meaningful stimuli. It is worthwhile to investigate whether or not other emotionally meaningful stimuli will achieve a better experimental result with our approach.

Among the 207 participants, healthy controls spent less time on the experimental paradigm than SCZ patients did, as illustrated in Figure 5b, because healthy controls are more flexible and have higher cognitive performance than SCZ patients (Queiros et al., 2019). Due to the differences in speech rates and ages of individual participants, there were inevitable differences in the recorded six time slots, which would directly affect our experimental results. To ensure the objectivity of experimental results, we did not intervene in their responses during the video acquisition process. In addition, our results indicate that the involvement of multiple specified questions in our experimental paradigm may be crucial (Table 3). However, the permutation and combination of selecting the six specified questions for experimental analyses led to multiple experimental results. Therefore, further research on high‐quality design is needed.

For the deep learning model, fixed architectures and iteration algorithms were used for the implementation of C‐CNN and FE‐CNN. The performance of C‐CNN and FE‐CNN depends largely on the architecture and training designs, including the number of layers, the selection of the appropriate spatial size of kernel and stride regions for convoluting and pooling layers, the optimal training parameters, the data augmentation strategy, and the iteration algorithm for the training process (Nanni et al., 2021). An in‐depth study could be introduced to evaluate the performance of C‐CNN and FE‐CNN with different architectures and training parameters. In addition, the use of FE‐CNN for classifying the labeled facial images in our own database was only 83.38% correct (Table 4). A more comprehensive facial expression database is required, which can be used for optimizing FE‐CNN training, and thus to classify correctly all facial images in our own database.

In the current study, besides of the aforementioned two uncontrollable factors, our sample is relatively small, which may lead to insufficient statistical power. All the above factors will be considered in the future to provide more powerful experimental results and findings.

## 5 CONCLUSION

We exploratively proposed a deep‐learning‐based approach to recognize SCZ patients using their facial images and further investigated the objective differences in several facial expressions between SCZ patients and healthy controls using a deep‐learning‐based approach. The testing data with the trained C‐CNN achieved high accuracy (95.18%) for the classification of “SCZ patient” and “healthy control.” Statistical results illustrated that there were significant differences in certain facial expressions between the two groups. The differences may be related to the underlying pathophysiological mechanisms and progression of SCZ. Although the analyses and discussions are preliminary, they will be of reference for us to carry on further study and research on facial expressions. We expect our approach can be applied to mobile devices for the aid–diagnosis of SCZ, and the facial expressions could be represented as clinical clues to help psychiatrists identify which people are at risk for developing mental disorders.

## AUTHOR CONTRIBUTIONS

Shen Li and Lili Wang supervised the study. Xiaofei Zhang and Xiaomei Shi conducted the diagnosis of SCZ and performed data acquisition. Tongxin Li and Xiaofei Zhang implemented the deep learning algorithm, conducted the data analyses, and drafted the manuscript. Shen Li assisted with writing and editing of the manuscript. Conghui Wang, Tian Tian, Haizhu Pang, and Jisong Pang performed the recruitment of participants. Jiangong Li, Lina Ren, Jing Wang, Lulu Li, and Yanyan Ma retrieved relevant literatures and summarized research outcomes. All authors have read and agreed to the published version of the manuscript.

## CONFLICT OF INTEREST STATEMENT

The authors declare no conflicts of interest.

### PEER REVIEW

The peer review history for this article is available at https://publons.com/publon/10.1002/brb3.3002.

## Supporting information

Supp Information

## Data Availability

The data that support the findings of this study are available on request from the corresponding author. The data are not publicly available due to privacy or ethical restrictions.
