# Supplementary material for: Recognizing schizophrenia using facial expressions based on convolutional neural network
Source: Brain Behav. 2023 Apr 16;13(5):e3002. doi: 10.1002/brb3.3002 (PMC10175991; doi:10.1002/brb3.3002)
Supplement: Supplementary file 1 — Supp Information [file BRB3-13-e3002-s001.docx]

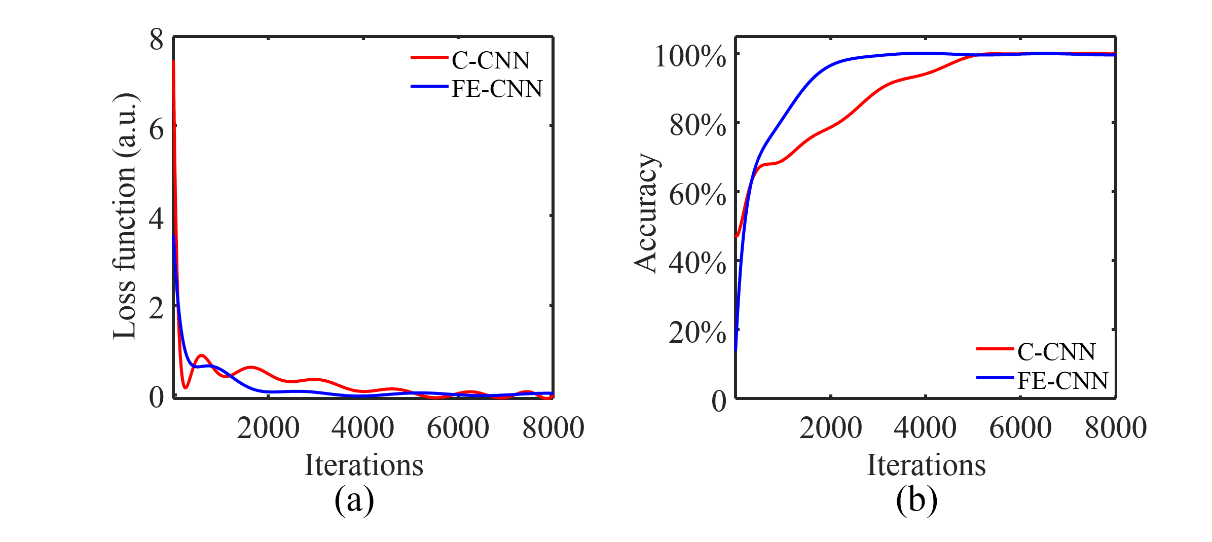


**SUPPLEMENTAL FIGURE 1** Training processes of C-CNN (e.g. K = 360) and FE-CNN: (a) the loss functions and (b) the accuracies as functions of iterations.


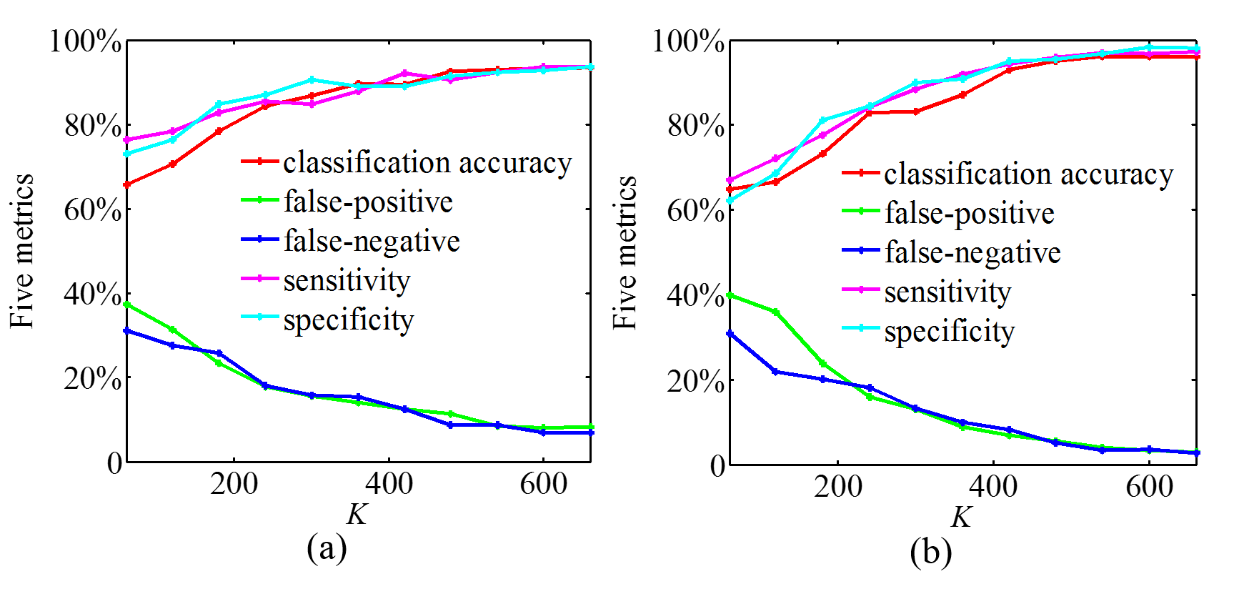


**SUPPLEMENTAL FIGURE 2** Quantitative evaluations for the classification of testing-data with the trained C-CNN under different amounts of input facial images, for the division of data-set with (a) traditional 7:3 and (b) 10-fold cross-validation methods.
